# Cranial Irradiation in Childhood Acute Lymphoblastic Leukemia Is Related to Subclinical Left Ventricular Dysfunction and Reduced Large Artery Compliance in Cancer Survivors

**DOI:** 10.3390/jcm8111952

**Published:** 2019-11-13

**Authors:** Joanna Sulicka-Grodzicka, Bernadeta Chyrchel, Justyna Totoń-Żurańska, Ewelina Nowak, Paweł P. Wołkow, Andrzej Surdacki, Tomasz Grodzicki

**Affiliations:** 1 Department of Rheumatology, Jagiellonian University Medical College, 10 Śniadeckich Street, 31-531 Cracow, Poland; 2 Second Department of Cardiology, Jagiellonian University Medical College, 17 Kopernika Street, 31-501 Cracow, Poland; chyrchelb@gmail.com (B.C.); surdacki.andreas@gmx.net (A.S.); 3 Center for Medical Genomics OMICRON, Jagiellonian University Medical College, 7C Kopernika Street, 31-034 Cracow, Poland; justyna.toton-zuranska@uj.edu.pl (J.T.-Ż.); pawel.wolkow@uj.edu.pl (P.P.W.); 4 Department of Internal Medicine and Gerontology, Jagiellonian University Medical College, 10 Śniadeckich Street, 31-531 Cracow, Poland; ewelina.nowak@uj.edu.pl (E.N.); tomekg@su.krakow.pl (T.G.)

**Keywords:** acute lymphoblastic leukemia, cardiovascular disease, childhood cancer survivors, cranial irradiation, echocardiography, systemic arterial compliance

## Abstract

Long-term survivors of acute lymphoblastic leukemia (ALL), the most common childhood malignancy, are at remarkably increased risk of heart failure (HF) in middle age, most likely due anthracycline cardiotoxicity. The role of cranial radiation therapy (CRT) in the development of left ventricular (LV) dysfunction, a predecessor of overt HF, remains unclear. Our aim was to compare LV function and systemic arterial properties according to past CRT in young adult survivors of anthracycline-treated ALL. We studied young adult survivors of childhood ALL at a median of 16 years from diagnosis treated with anthracycline-based chemotherapy, with (*n* = 12) or without (*n* = 30) CRT. In addition to fractional shortening (FS) and ejection fraction (EF), LV function was quantified by tissue Doppler imaging of the mitral annulus. Aortic strain/distensibility and arterial compliance were derived from echocardiography and simultaneously recorded pulse pressure. Despite similar FS and EF, peak mitral annular systolic velocity (median (interquartile range): 9.0 (7.5–10.0) vs. 10.0 (8.8–11.5) cm/s, *p* = 0.05), and early diastolic velocity (13.8 (13.0–14.8) vs. 15.5 (14.0–17.3), *p* = 0.01) were decreased after chemotherapy combined with CRT compared to chemotherapy without CRT. Systemic arterial compliance was lower in post-CRT subjects (1.0 (0.8–1.2 vs. 1.4 (1.1–1.7) mL/mmHg, *p* = 0.002). Aortic strain and distensibility were similar regardless of prior CRT. In conclusion, lower arterial compliance and subclinical LV dysfunction may be possible late consequences of past CRT in adult survivors of childhood ALL. Whether arterial stiffening is associated with future HF development in CRT-exposed ALL survivors remains to be investigated.

## 1. Introduction

Adult long-term survivors of childhood acute lymphoblastic leukemia (ALL), the most common childhood malignancy, are at risk of chronic health conditions, including atherosclerotic cardiovascular disease and heart failure (HF) [1,2,3]. Notably, the risk of developing symptomatic HF by age 40 years was elevated by 60–80%, even in low-risk subjects, from the Childhood Cancer Survivor Study (CCSS) in comparison with sibling controls, with a further increase in the mean relative risk exceeding 10 in higher-risk patients, identified on the basis of recognized risk factors for late HF [3].

The predictors of adult-onset HF in CCSS participants included younger age at malignancy diagnosis, cumulative anthracycline dose, concomitant cardiac irradiation, and female sex [3]. In another study cohort (DCOG-LATER study, Dutch Childhood Oncology Group–Long-Term Effects After Childhood Cancer), effects of earlier disease onset, increasing anthracycline dose, and cardiac irradiation were confirmed, with the cumulative incidence of developing severe HF 40 years after cancer diagnosis averaging 10.5% in the patients who had received only cardiotoxic chemotherapy (anthracycline, mitoxantrone, or cyclophosphamide), 27.8% for those with a history of both cardiotoxic chemotherapy and radiotherapy involving the heart, and only 0.3% in survivors without any past cardiotoxic treatment [4]. As the prevalence of asymptomatic left ventricular (LV) dysfunction, a predecessor of future overt HF, was 27% at a median of 15 years after diagnosis in adult childhood cancer survivors who had received any cardiotoxic treatment [5], the identification of determinants of subclinical LV dysfunction, preferentially with more sensitive novel echocardiographic techniques [6,7,8,9,10], is of practical relevance in terms of clinical surveillance and possible preventive measures.

Although the use of cranial radiation therapy (CRT) has considerably decreased over time, there are still many ALL survivors who have received prophylactic CRT based on the previous treatment protocols. In contrast to anthracyclines (initiating progressive loss of cardiomyocytes) and mediastinal irradiation (linked to myocardial fibrosis), CRT induces hypothalamic-pituitary dysfunction with consequent growth hormone (GH) deficiency and enhanced development of metabolic syndrome [11,12,13,14,15,16,17]. As early as 1993, Lehmann et al. were the first to describe depressed aortic distensibility in adults with GH deficiency [18]. Further studies confirmed elevated large artery stiffness in GH-deficient patients and demonstrated the ability of GH supplementation to improve systemic arterial compliance [19,20,21]. Additionally, Landy et al. observed that concomitant CRT was associated with additional decreases in LV mass and diastolic dimension in anthracycline-treated children (mainly with ALL) after a mean of 10 years from their diagnosis, in comparison with subjects after chemotherapy without CRT [22]. However, to the best of our knowledge, long-term joint effects of CRT and anthracycline exposure on large artery compliance have not been studied. Increased aortic pulse wave velocity and depressed systemic arterial compliance were previously demonstrated both early—already at 4-months after initiation of treatment with an anthracycline for various malignancies [23]—and late [24,25,26] in adolescent and young pediatric cancer survivors. 

Thus, we hypothesized that CRT can contribute to large artery stiffening in anthracycline-treated childhood-onset ALL subjects, thereby possibly potentiating associated risk of HF development in adulthood. Our aim was to compare estimates of LV function and systemic arterial properties according to past CRT in young adult survivors of childhood ALL.

## 2. Materials and Methods

### 2.1. Patients

Study participants included Polish adult childhood ALL survivors of Caucasian origin, diagnosed between the year 1994 and 2009. The survivor population was recruited from consecutive patients seen at the outpatient clinic at the University Hospital in Cracow from 2017 until 2018. We used medical records to obtain data on demographics, date of ALL diagnosis, the end of therapy, and treatment protocol including anthracycline exposure, CRT, and radiation dose (dose range: 12–18 Gy). Treatment regimens were based on protocols used in the treating institution at different time periods between the years 1994 and 2009.

Eligibility criteria included the following: Diagnosis of ALL before 18 years of age, five or more years since completion of cancer treatment, and exposure to anthracycline-based chemotherapy with or without CRT. Exclusion criteria encompassed the following: Time from the end of therapy for ALL shorter than 5 years, relapse or secondary cancer at the time of the study or during 5 preceding years, HF symptoms or HF-related medical therapy, any other self-reported cardioactive medication, coexisting diseases, or relevant abnormalities in routine blood and urine assays. ALL survivors treated with chemotherapy only were compared with those who had received the combination of chemotherapy with CRT.

The study was approved by the Bioethics Committee at the Jagiellonian University (approval No. 22.6120.274.2015). In agreement with the principles outlined in the 1975 Declaration of Helsinki, all participants provided written informed consent prior to the inclusion in the study. 

Study participants underwent a complex clinical evaluation including physical examination accompanied by anthropometric assessments, echocardiography, and blood sampling for future biochemical assays.

### 2.2. Echocardiographic Evaluation

Transthoracic echocardiography was performed by a well-trained experienced sonographer with Vivid 8 ultrasound equipment (GE Healthcare, Chicago, IL, USA). The sonographer was blinded regarding clinical data, including CRT exposure. Standard 2D-guided M-mode measurements of LV chamber dimensions and wall thickness were done in agreement with practice recommendations [27]. LV mass was calculated by means of the modified Devereux formula. LV ejection fraction (by the modified Simpson’s rule) and fractional shortening (from LV diameters in the parasternal long-axis view) were computed as indices of global LV performance [27].

Additionally, LV systolic and diastolic functions were estimated by tissue Doppler imaging, as follows: Mitral annular velocities were averaged from peak recordings taken at the lateral and septal region of the mitral annulus in systole (S’) and early diastole (e’) [9]. The peak early diastolic transmitral flow velocity (E) divided by averaged e’ (E-to-e’ ratio) was calculated as an index of left atrial (LA) pressure at the mitral valve opening [9,28]. The end-systolic cross-sectional area was computed for the left and right atrium, visualized in the apical 4-chamber view. The basal right ventricular (RV) transverse dimeter was measured in the RV-focused apical view, while the tricuspid annular plane systolic excursion (TAPSE) was measured in the apical 4-chamber view [27].

Aortic strain (Ao_strain_) was calculated as the percent difference between systolic (Ao_s_) and diastolic (Ao_d_) internal diameter (cm) of the ascending aorta, imaged 3 cm above the aortic valve in the left parasternal long-axis view, as follows: Ao_strain_ (%) = ((Ao_s_ − Ao_d_)/Ao_d_) × 100 [29,30]. Ao_s_ and Ao_d_ were measured at the maximal anterior motion of the aortic wall and at the R-wave peak on the simultaneous ECG tracing, respectively [30]. Aortic distensibility (Ao_dist_) was defined according to the following formula: Ao_dist_ (cm^2^ dyne^−1^) = 2 × (Ao_s_ − Ao_d_)/(Ao_d_ × (pulse pressure)) [29,30]. Pulse pressure was obtained by cuff sphygmomanometry of the brachial artery at the time of echocardiography [29,30], and a conversion factor of 1333 was applied to convert mm Hg into dynes/cm^2^. Finally, systemic arterial compliance (mL/mm Hg) was computed as stroke volume (derived from volumetric LV measurements) divided by the simultaneously recorded pulse pressure [31].

### 2.3. Biochemical Assays 

Fasting venous blood samples for biochemical assays were collected on the day of the echocardiographic assessment. Serum levels of glucose, high-density lipoprotein (HDL) cholesterol, total cholesterol, and triglycerides were measured (Cobas 6000/8000 analyzer, Roche Diagnostics, Indianapolis, IN, USA). Low-density lipoprotein (LDL) cholesterol concentrations were calculated using the Friedewald formula. N-terminal prohormone of B-type natriuretic peptide (NT-proBNP) was measured using a chemiluminescence immunoassay (Cobas E411 analyzer, Roche Diagnostics).

### 2.4. Statistical Analysis

We report data as means and SD, medians and interquartile range, or numbers. Patients’ characteristics were compared according to a history of past CRT by the Mann–Whitney U test, and Fisher’s exact test for continuous and categorical data, respectively. By a post-hoc analysis, the study design allowed the detection of intergroup differences of 0.95 SD (i.e., 2.2 cm/s for mitral S’, 3.7 cm/s for mitral e’, 2.2 × 10^−6^ cm^2^ dyne^−1^ for Ao_dist_, and 0.5 mL/mm Hg for arterial compliance) with a power of 80% at a type I error rate of 0.05. A *p*-value below 0.05 was inferred significant.

## 3. Results

Out of 60 potentially eligible subjects, 42 patients with complete data entered the study; the remainder either refused to participate or were eliminated on the basis of the exclusion criteria. The mean age of ALL survivors was 23 ± 4 years, and the time from ALL diagnosis averaged 16 ± 4 years. 

Patients’ clinical characteristics according to prior CRT, including traditional cardiovascular risk factors, were similar except for higher fasting glycaemia and shorter stature (by an average of 7 cm) in those with a history of CRT (Table 1).

Despite comparable LV fractional shortening and ejection fraction, longitudinal LV systolic function by tissue Doppler imaging was slightly, yet significantly, decreased in ALL survivors who had been treated with chemotherapy and CRT, in comparison with their counterparts who had received only chemotherapy (peak mitral annular systolic velocity: 9.0 (7.5–10.0) vs. 10.0 (8.8–11.5) cm/s, *p* = 0.05; early diastolic velocity: 13.8 (13.0–14.8) vs. 15.5 (14.0–17.3) cm/s, *p* = 0.01). Additionally, in patients with a history of past CRT, we observed a higher mitral peak E-to-e’ ratio, albeit within the normal range, while traditional diastolic estimates, including mitral inflow-based parameters and LA size, were unchanged (Table 2). 

With regard to vascular properties, systemic arterial compliance was lower in ALL survivors after CRT exposure (1.0 (0.8–1.2) vs. 1.4 (1.1–1.7) mL/mmHg, *p* = 0.002), whereas neither aortic strain (12.9 (5.9–19.0) vs. 9.3 (7.5–13.3) %), nor distensibility (3.1 (1.8–4.9) vs. 3.0 (2.0–4.5) (10^−6^ cm^2^ dyne^−1^)) differed by prior CRT (Table 2).

## 4. Discussion

Our principal finding was lower large artery compliance, with unchanged aortic strain and distensibility, and subclinical LV dysfunction revealed on tissue Doppler imaging in young adult survivors of childhood-onset ALL treated with both CRT and anthracycline-based chemotherapy in comparison with those who had received only chemotherapy. 

NT-proBNP levels did not differ significantly by prior CRT, which is in agreement with an improved ability of novel echocardiographic methods to better detect late subclinical LV dysfunction in asymptomatic childhood cancer survivors than natriuretic peptides assays [32,33,34]. Moreover, the superiority of novel echocardiographic techniques, i.e., global longitudinal strain analysis and tissue Doppler imaging, over conventional parameters to detect systolic [7,8] and diastolic [6,10] LV dysfunction has previously been demonstrated in long-term survivors of childhood cancer, mainly after ALL. However, those studies [6,7,10] reported additive contributions of the cumulative dose of anthracyclines and mediastinal irradiation, but not CRT, to subclinical LV dysfunction about 20 years after malignancy diagnosis. Of note, compared to their counterparts, LV mass was similar and LV size was smaller in our post-CRT subjects, in partial contrast to the study by Landy et al., who observed associations of CRT exposure with lower both LV dimension and mass, but unchanged LV systolic function in anthracycline-treated childhood cancer survivors [22]. Nevertheless, in that study LV diastolic function was not estimated and LV fractional shortening was the only parameter of LV performance. 

As to earlier studies focused on arterial properties in anthracycline-treated childhood cancer survivors, none of those reports has linked elevated arterial stiffness to prior CRT [24,25,26]. Thus, our publication is the first report of decreased systemic arterial compliance and subclinical LV dysfunction as a possible late consequence of past CRT in adult ALL survivors. Hummel et al. demonstrated subtle LV systolic and diastolic dysfunction, despite unchanged EF, at a median of 21.7 years follow-up in a small group of survivors of head and neck cancer treated with cranial and neck radiotherapy without additional systemic therapy, however the radiation dose was considerably higher in this group compared to CRT dose in ALL [35]. Furthermore, data from pediatric differentiated thyroid carcinoma survivors suggest that consequences of treatment with thyroidectomy and radioiodine may as well have delayed effects on the heart, including subclinical LV dysfunction [36]. 

Admittedly, on the basis of our small cross-sectional study, we can only speculate on the potential causal mechanisms of these observations. However, there is some evidence that the interference of CRT with the hypothalamic-pituitary function with subsequent GH deficiency and insulin resistance can underlie our findings because a history of past CRT was associated with marginally higher fasting glycaemia and shorter stature in the ALL survivors participating in the present study. 

Firstly, the prevalence of metabolic syndrome was over 3-fold higher (23% vs. 7%) in ALL survivors treated with CRT and chemotherapy compared to those who had received only chemotherapy [16]. Similar relative differences (60% vs. 20%) were earlier reported for the prevalence of two or more of the five classical diagnostic components of the metabolic syndrome in ALL survivors [13]. Secondly, not only was the magnitude of insulin resistance and prevalence of metabolic risk factors increased after past chemotherapy combined with CRT, but, notably, 85–91% of those subjects were GH-deficient [12,13], and two-year GH replacement therapy attenuated metabolic abnormalities and improved LV systolic function [17]. Thirdly, GH supplementation improved depressed arterial compliance in GH-deficient adults [19,20,21]. 

Therefore, our results may be suggestive of site-specific vascular effects of CRT-dependent GH deficiency and consequent insulin resistance. In 518 asymptomatic young adults participating in the Bogalusa Heart Study, the magnitude of insulin resistance (by the homeostasis model assessment) was associated with lower large artery but not small artery compliance, also including the arteriolar bed [37]. Moreover, endothelium-dependent responses of small resistance vessels were not improved after GH supplementation in GH-deficient subjects, despite a simultaneous improvement in large artery compliance [19,20,21]. Lastly, in former ALL patients who had survived, on average, almost 25 years from disease onset, Dengel et al. demonstrated that CRT added to conventional chemotherapy was not associated with a further exacerbation of endothelial-dependent flow-mediated dilation of the brachial artery in comparison with those who had received only chemotherapy [38]. 

That large-artery compliance reflects distinct vascular properties, not covered by investigations focused on the distal part of circulation or endothelial function, may translate into different pathogenic relevance of respective parameters derived from various techniques of vascular studies. In particular, in young adult ALL survivors, endothelial dysfunction, reported previously by several research groups, including ours [24,38,39,40,41], appears to be a hallmark of accelerated atherogenesis and an antecedent of plaque formation, while large artery stiffening reflects impaired ventriculoarterial coupling.

As the pressure wave propagates faster in the stiffened arteries, it returns earlier to the heart, thereby elevating central systolic blood pressure, despite unchanged peripheral blood pressure, as in our study subjects. This results in increased LV afterload and a prolonged LV ejection period with concomitant reduction of LV filling time and diastolic pressure, governing coronary blood supply to the LV myocardium. Aortic compliance increases during the first decade of life and reaches a peak around puberty [42]. Consequently, factors affecting vascular structure during middle childhood and early adolescence, such as exposure to chemotherapy and CRT for ALL, are likely to translate into impaired large artery compliance and excessive LV afterload in adulthood. 

An increased pressure load on the LV myocardium in combination with compromised coronary flow predominantly affects longitudinally oriented subendocardial myocardial fibers, which translates into a selective impairment of LV long-axis function despite unchanged global LV systolic performance, a feature of HF with preserved ejection fraction [43]. Estimates of LV longitudinal function are the preferred parameters for early detection of cardiotoxicity in long-term cancer survivors before detectable changes in classical measures of LV function [6,7,8]. That our ALL survivors with a history of past CRT presented with lower maximal systolic and early diastolic velocity of the mitral annulus along the longitudinal direction in addition to slightly higher mitral peak E-to-e’ ratio, an index of LV filling pressure at mitral valve opening, is consistent with an initial stage of LV dysfunction, keeping in mind unchanged LV fractional shortening, ejection fraction, LA size, and mitral E/A ratio.

Our findings should be interpreted with caution in terms of their potential clinical significance. We may only hypothesize that subclinical late LV dysfunction after anthracycline-based chemotherapy could possibly be enhanced by slowly-developing arterial stiffening attributable to past CRT. Curiously, in an animal model of late-occurring anthracycline-induced cardiac dysfunction, prior doxorubicin exposure abolished LV hypertrophy in response to angiotensin II-induced hypertension [44]. Since LV hypertrophy is perceived as a compensatory mechanism triggered by excessive LV afterload, anthracycline-based chemotherapy can also block an appropriate cardiac adaptation to LV pressure overload evoked by depressed systemic arterial compliance in those with a history of concomitant CRT. Therefore, since hypertension predisposes to anthracycline-induced cardiotoxicity [45] and is the most potent modifiable risk factor for overt HF in long-term childhood cancer survivors [46], our results may reflect an earlier phase of this interaction, i.e., association between subclinical LV dysfunction, a HF antecedent, with accelerated arterial stiffening, a predictor of incident hypertension [47]. As patients exposed to even low doses of anthracyclines may show subclinical cardiac abnormalities [48,49], further studies are warranted in search of modulators of the risk of late cardiotoxicity in childhood cancer survivors.

### Limitations

Firstly, our study is limited by a small number of patients and a retrospective design, thus the preliminary results require confirmation in large cohorts of ALL survivors. Secondly, longitudinal LV systolic function was estimated only by tissue Doppler imaging of the mitral annulus, while longitudinal strain analysis by speckle-tracking echocardiography has been considered the best method to detect subtle chemotherapy-related LV dysfunction [8,50]. Nevertheless, in the case of unavailability of speckle-tracking echocardiography, the quantification of mitral annular velocity by tissue Doppler imaging is also recommended for this purpose [8]. Thirdly, biochemical characteristics of the study subjects included only basic laboratory parameters. In particular, GH secretion, insulin-like growth factor-1 levels, and the magnitude of insulin resistance have not been measured, which limits mechanistic interpretations of our findings. Likewise, we have estimated only NT-proBNP, whereas other circulating markers were previously shown to independently correlate with the magnitude of myocardial fibrosis in HF with preserved ejection fraction [51]. Finally, the absence of coexistent diseases and cardiovascular medications was established mainly on the basis of patients’ self-reported histories. On the other hand, we have applied a wide set of exclusion criteria to ensure the homogeneity of the study group.

## 5. Conclusions

Our preliminary study appears to suggest decreased large artery compliance and subclinical LV dysfunction as possible late consequences of concomitant past CRT in young adult survivors of childhood-onset anthracycline-treated ALL. Whether arterial stiffening can contribute to LV dysfunction and late development of overt HF in CRT-exposed ALL survivors, remains to be investigated.

## Figures and Tables

**Table 1:** Clinical characteristics of survivors of childhood acute lymphoblastic leukemia (ALL) according to past cranial radiotherapy (CRT) in addition to anthracycline-based chemotherapy.

Characteristic	Treatment for Childhood-Onset ALL		*p*-Value
	Chemotherapy with CRT	Chemotherapy without CRT	
Age, year	23 (20–25)	22 (20–25)	NS
Men/Women	6/6	15/15	NS
Time from ALL diagnosis, years	15 (10–18)	17 (12–19)	NS
Height, cm	165 (155–176)	171 (164–180)	0.09
Body-mass index (kg/m^2^)	24.0 (22.1–26.4)	23.2 (21.0–26.0)	NS
Waist-to-hip ratio	0.81 (0.80–0.89)	0.81 (0.76–0.84)	NS
Heart rate, beats/min	74 (68–86)	76 (70–85)	NS
Systolic blood pressure, mm Hg	119 (112–135)	126 (113–128)	NS
Pulse pressure, mm Hg	55 (48–63)	49 (39–57)	0.14
eGFR, mL/min per 1.73 m^2^	126 (116–131)	121 (113–128)	NS
Glucose, mmol/L	4.9 (4.6–5.2)	4.7 (4.5–4.9)	0.07
Cholesterol, mmol/L	4.5 (3.9–5.0)	4.2 (3.8–4.8)	NS
HDL cholesterol, mmol/L	1.8 (1.2–2.3)	1.7 (1.4–1.9)	NS
LDL cholesterol, mmol/L	2.1 (1.8–2.5)	2.3 (1.8–2.6)	NS
Triglycerides, mmol/L	0.8 (0.6–1.3)	0.8 (0.6–1.1)	NS
NT-proBNP, pg/mL	50.6 (18.2−71.6)	26.0 (16.7−56.7)	NS

Data are shown as median (interquartile range) or numbers. eGFR, estimated glomerular filtration rate by the CKD-EPI equation; HDL, high-density lipoprotein; LDL, low-density lipoprotein; NS, non-significant; NT-proBNP, N-terminal prohormone of B-type natriuretic peptide.

**Table 2:** Echocardiographic characteristics of survivors of childhood acute lymphoblastic leukemia (ALL) according to past cranial radiotherapy (CRT) in addition to anthracycline-based chemotherapy.

Characteristic	Treatment for Childhood-Onset ALL		*p*-Value
	Chemotherapy with CRT	Chemotherapy without CRT	
LVd, mm	44 (40–48)	48 (44–51)	0.03
PWd, mm	8 (7–9)	8 (7–8)	NS
IVSd, mm	8 (7–9)	8 (7–9)	NS
LV mass index, g	55 (44–65)	59 (51–64)	NS
LV ejection fraction, %	65 (59–68)	65 (64–70)	NS
LV fractional shortening, %	35 (32–39)	36 (34–42)	NS
LA-csa, cm^2^	15.0 (12.2–20.0)	16.8 (14.5–18.5)	NS
RVd, mm	34 (31–36)	32 (31–36)	NS
TAPSE, mm	27 (23–27)	26 (25–28)	NS
RA-csa, cm^2^	11.5 (10.4–15.4)	12.0 (11.0–14.1)	NS
Mitral peak E, cm/s	99 (85–100)	90 (80–100)	NS
Mitral peak E/A ratio	1.8 (1.4–2.1)	2.0 (1.5–2.8)	NS
Mitral peak e’, cm/s	13.8 (13.0–14.8)	15.5 (14.0–17.3)	0.01
Mitral peak E/e’ ratio	6.7 (5.7–7.3)	5.9 (5.2–6.9)	0.10
Mitral peak S’, cm/s	9.0 (7.5–10.0)	10.0 (8.8–11.5)	0.05
Aortic diastolic diameter, mm	25 (21–30)	26 (24–27)	NS
Aortic strain, %	12.9 (5.9–19.0)	9.3 (7.5–13.3)	NS
Aortic distensibility, 10^−6^ cm^2^ dyne^−1^	3.1 (1.8–4.9)	3.0 (2.0–4.5)	NS
Arterial compliance, mL/mm Hg	1.0 (0.8–1.2)	1.4 (1.1–1.7)	0.002

Data are shown as median (interquartile range). Bold values indicate a *p*-value <0.05. A, late diastolic inflow velocity; E, early diastolic inflow velocity; e’, early diastolic annular velocity averaged from measurements taken at the lateral and septal region of the mitral annulus; IVSd, end-diastolic interventricular septum thickness; LA-csa, end-systolic left atrial cross-sectional area in the apical 4-chamber view; LV, left ventricular; LVd, end-diastolic LV diameter in the parasternal long-axis view; NS, non-significant; PWd, end-diastolic LV posterior wall thickness; RA-csa, end-systolic right atrial cross-sectional area in the apical 4-chamber view; RVd, basal right ventricle (RV) transverse dimeter in the RV-focused apical view; S’, systolic annular velocity averaged from measurements taken at the lateral and septal region of the mitral annulus; TAPSE, tricuspid annular plane systolic excursion in the apical 4-chamber view.
